# Advances in fetal surgery

**DOI:** 10.1590/S1679-45082016MD3449

**Published:** 2016

**Authors:** Denise Araujo Lapa Pedreira

**Affiliations:** 1 Universidade de São Paulo, São Paulo, SP, Brazil.

**Keywords:** Fetus/surgery, Fetoscopy/methods, Minimally invasive surgical procedures, Meningomyelocele, Spinal dysraphism

## Abstract

This paper discusses the main advances in fetal surgical therapy aiming to inform health care professionals about the state-of-the-art techniques and future challenges in this field. We discuss the necessary steps of technical evolution from the initial open fetal surgery approach until the development of minimally invasive techniques of fetal endoscopic surgery (fetoscopy).

## INTRODUCTION

Currently, a number of fetal malformations can be treated with fetal surgery, including monochorionic twin gestation complications (twin transfusion syndrome, acardiac twin, isolated intrauterine growth restriction, etc.), congenital diaphragmatic hernia (an intratracheal balloon is placed using fetal bronchoscopy), constrictive amniotic bands, lower urinary tract obstruction^1^ and, more recently, myelomeningocele.

Fetal surgery began in the 1980s via open surgery (maternal laparotomy, followed by hysterectomy with direct exposure of the fetus) and was gradually replaced by a less invasive surgical technique named fetoscopy, where ultrasound guides the entrance of a videocamera inside the uterus. In the beginning, fetoscopy was carried out only in amniotic fluid medium, using a single port to access the uterine cavity and using an endoscopic scope with a working channel where a laser fiber can be fitted for the coagulation of blood vessels, where micro catheters go through for the balloon insertion, as well as, small bipolar forceps.^2,3^

However, the fluid medium poses limitations for more complex surgeries that require dissection and suture. Images acquired in fluid medium have lower quality than in the aerial medium and, if bleeding occurs, the hemorrhagic fluid does not allow an adequate imaging, and it can prevent the procedure to be completed. Other limitation is the “fluctuation” of the fetus that leaves the ideal position. Therefore, to perform fetoscopy in the aerial medium became crucial to the advances in fetal surgery.

Animal model studies carried out in 1990s showed the occurrence of fetal acidosis due to the insufflation of carbonic gas in the uterine cavity^4^ and, for some years, its applicability was limited. In 2010 Kohl et al.^5^ showed the absence of changes in the anatomy of the central nervous system due to the use of CO2 in sheep fetuses submitted to fetoscopy. In their study low insufflation pressure was used and there was only partial carbon dioxide insufflation of amniotic cavity which was left with some amount of amniotic fluid. Procedures were completely percutaneous without removal of uterus from the abdominal cavity, this constitutes an important difference in relation to the previous studies. We believe that taking the uterus out, *per se*, can compromise its irrigation (compression of vascular pedicle), additionaly leading to hyperinsufflation of the thin sheep’s uterus. So, both situations can potentially impair the uterus-placenta circulation.

In 2011, a prospective and randomized study was published,^6^ comparing the prenatal repair of myelomeningocele with postnatal correction. The study showed that fetuses submitted to antenatal repair had 50% less need of ventriculoperitoneal shunting to treat hydrocephalus, in addition they have twice the chance to ambulate without any support. This study, Management of Myelomeningocele Study (MOMS), used open surgery for fetal repair, which established the maternal risks associated of using this route, including acute pulmonary edema after surgery, need of blood transfusion and unfavorable uterine scarring (dehiscence or very thin scar) of the surgical site, in approximately 35% of cases.

In open surgery, the laparotomy is wide and normally requires the sectioning of maternal abdominal muscles, because the uterus needs to be removed from the abdominal cavity. Different from the c-section hysterotomy, the open fetal surgery approach, does not respect the uterine segment region, where muscle fibers have more parallel orientation, potentially preserving the myometrium. In open surgery, hysterotomy is performed in the second trimester, when the uterine segment is still not well defined, and must be done in the best area to access the fetal spine, and to avoid the placental insertion site. This area is normally found in the body or fundal uterine region, where the muscle fibers have an helicoidal distribution (in order to enable the physiologic “triple gradient” of uterine contraction). Therefore, even a smaller size hysterotomy potentially carries the same risks, also this abnormal scarring generates a zone of weakness that contraindicates the labor in both current or future gestations. There are reports of uterine rupture after fetal open surgery that occur in the second trimester, in the absence of contractions, caused only by the uterine distension, which is a risk to the mother and fetus. After a c-section, hysterorrhaphy can heal without tension, because the baby is already out, while in the open surgery, the fetus remains and continues to growth – therefore, the hysterorrhaphy remains under constant and progressive tension.

Despite these risks, open fetal surgery became the gold-standard to treat myelomeningocele and the seeking for minimally invasive techniques to increase maternal safety became the current challenge in fetal therapy surgery.

Currently, only two groups have pursued an entirely percutaneous endoscopic approach for the prenatal treatment of myelomeningocele. One group from Germany, Kohl et al.^7^ and, one from Brazil, Pedreira et al.^8^, both groups use fetoscopy with partial carbon dioxide insufflation. But different surgical techniques for the repair itself. Similarly, to the transition between performing surgery using laparotomy to using the laparoscopic approach, it was necessary to develop new surgical techniques, new instruments, trocars access, closure devices, etc.

Recent studies have shown that the German technique^9^ has achieved neurological developmental results that are quite similar to the results of the MOMS’s study, but with minimal maternal morbidity. The Brazilian technique, (SAFER - Skin-over-biocellulose for Antenatal Fetoscopic Repair) has obtained superior neurologic results compared to the MOMS’s study, but these results are preliminary (23 operated on cases so far). However, because three ports are need to access the uterine cavity, the mean gestational age of delivery is slightly inferior, and the premature rupture of membrane rate is superior than the results of MOMS´s study. We believe that further technical development in the near future, will confirm if this new technique is not only SAFER to the mother, but also better to the fetus. A work coming from Brazil that may shape a technique to be applied worldwide in the future.
