# Supplementary material for: Advances in fetal surgery
Source: Einstein (Sao Paulo). 2016 Jan-Mar;14(1):110–2. doi: 10.1590/S1679-45082016MD3449 (PMC4872928; doi:10.1590/S1679-45082016MD3449)
Supplement: Translation

# Translation: Avanços em cirurgia fetal

**Authors:** Denise Araujo Lapa Pedreira

**Affiliations:** 1 Universidade de São Paulo, São Paulo, SP, Brasil.

**Keywords:** Feto/cirurgia, Fetoscopia/métodos, Procedimentos cirúrgicos minimamente invasivos, Meningomielocele, Disrafismo espinal

## Abstract

São discutidos os principais avanços na terapia cirúrgica do feto, tendo como objetivo informar profissionais da área da saúde sobre qual o estado atual da arte e quais seus desafios futuros. São debatidos os passos que foram necessários para a evolução da técnica inicial, quando a cirurgia fetal era realizada a céu aberto, até o desenvolvimento de técnicas minimamente invasivas, de cirurgia endoscópica fetal (fetoscopia).

## INTRODUÇÃO

Atualmente, várias malformações fetais podem ser tratadas por cirurgia fetal, a saber: complicações da gestação gemelar monocoriônica (transfusão feto-fetal, gêmeo acárdico, crescimento intrauterino retardado isolado etc.), hérnia diafragmática fetal (por meio de broncoscopia fetal e colocação de balão intratraqueal), brida amniótica constritiva, obstruções urinárias baixas^1^ e, mais recentemente, a meningomielocele.

O tratamento cirúrgico do feto, iniciado na década de 1980, por meio da via a céu aberto (laparotomia materna, seguida de histerotomia e exposição fetal direta), vem sendo gradativamente substituído por uma técnica menos invasiva, na qual através da ultrassonografia se insere uma câmera dentro do útero, denominada fetoscopia. Inicialmente a fetoscopia era realizada apenas em meio líquido (líquido amniótico), utilizando-se um único orifício de entrada para acessar a cavidade uterina, por onde se insere uma ótica com canal de trabalho, para passagem de fibra de laser para coagulação de vasos, micro cateteres para passagem de balão e pequenas pinças bipolares.^2,3^

No entanto, o meio líquido apresenta limitações para a realização de cirurgias mais complexas, que exigem dissecção e sutura. A imagem obtida em meio líquido tem qualidade inferior ao meio aéreo e, caso ocorra sangramento, o líquido hemorrágico não permite uma visualização adequada, podendo impedir a conclusão do procedimento. Outra limitação é a “flutuação” do feto, que sai da posição ideal. Dessa forma, realizar a fetoscopia no meio aéreo tornou-se imperativo para o avanço da cirurgia fetal.

Estudos em modelo animal, realizados no final da década de 1990, demonstraram a ocorrência de acidose fetal consequente à insuflação de gás carbônico na cavidade uterina^4^ e, durante alguns anos, seu emprego foi limitado. Foi apenas em 2010 que Kohl et al.^5^ demonstraram ausência de alterações do desenvolvimento neurológico ou anatômicas do sistema nervoso central, consequentes à exposição ao gás carbônico em fetos de ovelha submetidos a fetoscopia. Neste estudo, foram utilizadas baixa pressão do gás e insuflação parcial da cavidade amniótica, deixando alguma quantidade de líquido amniótico no útero. Os procedimentos foram inteiramente percutâneos, sem a retirada do útero de dentro da cavidade abdominal, que constitui diferença importante em relação aos estudos anteriores. Acredita-se que a exteriorização do útero, *per se*, possa comprometer sua irrigação (compressão do pedículo vascular), além da possibilidade de ocorrer hiperinsuflação do útero muito fino da ovelha − nos dois casos potencialmente prejudicando a circulação útero-placentária.

Em 2011, foi publicado um estudo prospectivo e randomizado,^6^ comparando a correção pré-natal da mielomeningocele com a correção após o nascimento, no qual foi demonstrado que os fetos submetidos à correção antenatal tinham 50% menos necessidade de derivação ventrículo-peritoneal para tratar a hidrocefalia, além do dobro da possibilidade de deambular sem qualquer auxílio. Este estudo, denominado *Management of Myelomeningocele Study* (MOMS) utilizou a via a céu aberto para correção fetal, ficando bem demonstrados os riscos maternos associados à utilização dessa via, a saber: edema agudo de pulmão após a cirurgia, necessidade de transfusão de sangue no parto e cicatrização uterina desfavorável (deiscência ou cicatriz muito fina) no local da cirurgia em, aproximadamente 35% dos casos.

Na cirurgia a céu aberto, a laparotomia é ampla, normalmente seccionando a musculatura abdominal materna, pois o útero precisa ser luxado para fora da cavidade abdominal. Diferente da cesárea, a histerotomia que é realizada para a cirurgia fetal não respeita a região do segmento uterino, onde as fibras musculares têm orientação mais paralela, potencialmente poupando o miométrio. Na cirurgia a céu aberto, a histerotomia é realizada no segundo trimestre, quando o segmento uterino ainda não está bem definido, e na melhor área para acessar a coluna do feto, evitando a inserção placentária. Isso normalmente se encontra na região corporal ou fúndica, onde as fibras musculares têm distribuição helicoidal (para permitir o “tríplice gradiente” fisiológico da contração uterina). Desse modo, por menor que esta histerotomia seja, é gerada uma zona de fragilidade que contraindica o trabalho de parto, tanto na gestação em curso, quanto em futuras gestações. Existem relatos de rotura uterina, após cirurgia fetal a céu aberto, que ocorreram no segundo trimestre, mesmo fora do trabalho de parto, apenas pela distensão do útero numa gestação subsequente, o que coloca mãe e feto em risco. Na cesárea, a histerorrafia pode cicatrizar sem tensão, já que o parto é realizado, enquanto na cirurgia a céu aberto, o feto permanece e continua crescendo − portanto, a histerorrafia fica sob constante e progressiva tensão.

Apesar desses riscos, a cirurgia fetal tornou-se o padrão-ouro para o tratamento da mielomeningocele, e a busca por técnicas minimamente invasivas para aumentar a segurança materna tornou-se o desafio atual na terapia cirúrgica fetal.

Atualmente, apenas dois grupos realizam a cirurgia fetal endoscópica inteiramente percutânea, para tratamento da meningomielocele. Na Alemanha, o grupo de Kohl et al.^7^ e, no Brasil, o grupo de Pedreira et al.,^8^ ambos utilizando a fetoscopia com insuflação parcial de gás carbônico (PCI - *partial carbono dioxide insuflation*). Os grupos usam técnicas diferentes para a correção do defeito propriamente dito. Pois, como ocorreu na transição entre a cirurgia realizada por laparotomia e a cirurgia laparoscópica, foi necessário desenvolver novas técnicas cirúrgicas, instrumentos, trocaters de acesso, dispositivos de fechamento, etc.

Resultados recentes têm demonstrado que a técnica Alemã^9^ alcança resultados neurológicos bastante semelhantes ao do estudo MOMS, com mínima morbidade materna. A técnica brasileira, denominada (SAFER - *Skin-over-biocellulose for Antenatal FEtoscopic Repair*), tem obtido resultados neurológicos, ainda que preliminares (23 casos operados até o momento), superiores aos do estudo MOMS. No entanto, por necessitar de três orifícios para acesso da cavidade uterina, a idade gestacional média do parto ainda tem sido inferior, e a rotura prematura de membranas, superior, quando comparadas a este estudo. Acreditamos que, com um maior desenvolvimento técnico, em um futuro breve, essa nova técnica possa se demonstrar não apenas mais segura para a mãe, mas ainda melhor para o feto. Ventos que sopram do nosso país... para moldar a técnica que será empregada no futuro em todo o mundo.
